# A Cross-Sectional Study on Balance Deficits and Gait Deviations in COPD Patients

**DOI:** 10.1155/2021/6675088

**Published:** 2021-01-06

**Authors:** Priyanka Jirange, K. Vaishali, Mukesh Kumar Sinha, Kalyana Chakravarthy Bairapareddy, Gopala Krishna Alaparthi

**Affiliations:** ^1^Department of Physiotherapy, Manipal College of Health Professions, Manipal Academy of Higher Education, Manipal 576104, Karnataka, India; ^2^Department of Physiotherapy, College of Health Sciences, University of Sharjah, Sharjah, UAE

## Abstract

**Background:**

The gait abnormalities were linked to the balance deficits in the previous studies. However, the deviations in the gait parameters in COPD are currently not known. The study aims to compare gait parameters, static and dynamic balance, and risk of falls in COPD with those in non-COPD individuals.

**Method:**

Fourty-two patients with COPD aged 45 years and gender-matched control subjects were included in the study. Gait parameters were assessed by Win-Track gait analyzer, the static balance was assessed by posturography, and the dynamic balance was assessed by the time up and go test. The fear of falls was assessed by Falls Efficacy Scale.

**Results:**

COPD individuals had decreased static and dynamic balance as assessed by posturography (*p* < 0.05) and TUG (*p* < 0.01), respectively. A significant difference in swing duration (*p*=0.004) and also increased risk of falls (*p* < 0.01) was observed in COPD patients as compared to non-COPD individuals.

**Conclusion:**

COPD individuals have increased swing duration, reduced static and dynamic balance, and increased fear of falls as compared to non-COPD individuals.

## 1. Introduction

COPD is forecast to become the world's third major cause of death in the global disease burden by 2030 [1, 2]. The 2016 Global Disease Burden Study reports a prevalence of 251 million COPD instances worldwide [3]. According to a 2006 multicentre research sponsored by the Indian Medical Research Council (ICMR), the prevalence of COPD in people over 35 years in India is 4.1 percent [4].

Chronic obstructive pulmonary disease (COPD) is a preventable and treatable disease with several extrapulmonary manifestations, including cachexia and skeletal muscle dysfunction, contributing to morbidity and mortality [5, 6]. It has been well established that skeletal muscle function (strength and endurance) and structure (fiber size, fiber-type distribution, capillary density, and metabolic capacity) are altered in patients with COPD, leading to impaired balance and mobility [6, 7]. Balance and mobility are significant aspects of most daily living activities, and studies have shown that decreased muscle power and hypoxia affect static and dynamic balance in COPD patients [8, 9]. Balance impairment is associated with an increased risk of falling with a rise in adult morbidity rate [10]. A cross-sectional study revealed that COPD is the most prevalent chronic condition with the highest prevalence of falls, second only to osteoarthritis [11].

In COPD patients, reduced lower limb function and balance deficit may compromise the stability needed for walking, thus altering gait in COPD patients compared to healthy elderly patients [12, 13]. However, the variations in the spatiotemporal gait parameters in COPD patients are presently not known. The objective of the study was to compare gait parameters, static and dynamic balance, and risk of falls in COPD with those in non-COPD individuals.

## 2. Methods

The study protocol has been evaluated and endorsed by the institutional research committee and university ethical committee. All subjects were notified of the study's purpose and procedure. In a cross-sectional study, 42 patients aged 45 years and diagnosed with COPD and gender-matched individuals with no history of COPD (non-COPD) were recruited. Patients between 50 and 60 years of age with medically stable COPD were categorized according to GOLD criteria. The research respondents who had history of orthopaedic, neurology, central or peripheral vestibular illnesses, and diabetic neuropathy were excluded. Age- and gender-matched healthy subjects were recruited during the camps as part of the community screening. Individuals who were willing to participate and gave written informed consent were part of the study. For all respondents, weight, height, and body mass index were recorded as part of the initial assessment. COPD patients were tested for pulmonary function, and the severity of the disease was investigated.

## 3. Gait Evaluation

Using the Win-Track gait analyser, gait parameters were evaluated. It comprises a 1616 mm long platform with a width of 652 mm and a height of 30 mm [14]. Three trails were given to all the participants and were instructed to walk on the platform with at least three steps. Three-step gait protocol was used as it had shown good reliability in measuring temporal variables [14]. Subjects were advised to look forward and walk on the platform at a comfortable pace. The data were uploaded to a computer for further quantitative analysis.

## 4. Balance Assessment

The assessment of static balance was done by posturography (Good BalanceTM, Metitur Ltd., Finland) [15, 16]. The participants were made to stand on the platform of posturography and look for 30 seconds at a fixed point on the opposite wall. For the eye-open on foam (EOF) and eye-closed on foam (ECF) conditions, the participants stood on a 2.5 cm thick piece of foam placed on the top of the force plate. The postural sway was observed with eyes open and closed on stable surfaces on the force platform in normal standing.

## 5. Timed Up and Go (TUG) Test

To evaluate the dynamic balance, the TUG test was performed. For the evaluation, a chair with armrest, a stopwatch, and an inch tape were used to mark the distance of three meters. A practice session was conducted before the timed test. The participants sat down and were requested to stand up, walk three meters, turn around, walk back to the chair, and sit down. The time taken to complete the test was recorded, and the 14-second cutoff value was used to determine the risk of falling [17, 18].

## 6. Fear of Falls Assessment

Falls Efficacy Scale (FES) questionnaire was used to evaluate the fear of falling or continuous worry about falling that may restrict daily living activities. The participants were asked to rate on a four-point Likert scale about the possibility of falling while performing 16 different activities. A high score on the total score of 28–64 in FES-I scale (PRoFaNE group) indicates the high concern about falling [19].

## 7. Data Analysis

Data were analysed using SPSS version 15. Test for normality was done. Descriptive statistics were used to analyse the demographic data. Independent *t*-test was used for the comparison of demographic data. Mann–Whitney *U*-test was used to compare the gait parameters, posturography values, scores of Falls Efficacy Scale, and time of TUG test between COPD and healthy subjects. Spearman's rank correlation coefficient was used to determine the correlation between Fev1% and balance parameter for COPD participants. The level of significance was set at *p* < 0.05.

## 8. Results

The static balance, gait assessment and TUG tests were successfully performed by both groups of people without losing balance. The body mass index was comparable between the two groups. The characteristics of the subjects are presented in Table 1. No significant difference was observed in any demographic variable. COPD individuals have increased swing duration in comparison with non-COPD individuals with *p* < 0.05 shown in Table 2. The findings of both COPD and non-COPD posturography measures are summarised in Table 3. In contrast to non-COPD individuals, all posturographic variables indicating more postural sway in COPD individuals, however, NSEC-AP, ML sway, and NS EO-AP sway are statistically significant (*p* < 0.05). In addition, the velocity moment was found to be more in COPD individuals compared to non-COPD individuals, although NSEO-VM is statistically significant (*p* < 0.05). The outcome of the FES-I score is also shown in Table 3. Individuals with COPD reported higher FOF with a median of 32 (24.75–36) compared to non-COPD individuals. There was no significant correlation between FEV1 and balance parameters in COPD patients (Table 4).

## 9. Discussion

The static, dynamic balance, gait parameters, and fear of falling were significantly different in COPD individuals compared to age- and gender-matched non-COPD individuals.

It was found that the swing time in COPD individuals was significantly affected. The findings are consistent with a study by Yantes et al. [20, 21] which noted biomechanical gait alterations in COPDs. Another study by Annegarn et al. [13] showed that subjects with COPD walked at a decreased speed, decreased cadence, and greater medium-lateral variability during 6MWT compared to healthy elderly subjects measured by triaxial accelerometers. From the clinical point of view, reduced physical activity in daily life, impaired muscle strength, altered breathing dynamics, and poor balance are the mostly likely causes for gait abnormalities in COPD individuals [20]. Decreased levels of physical activity result in decreased muscle fiber cross-sectional area and reduction in mitochondrial density leading to skeletal muscle dysfunction which is commonly seen in COPD individuals, and these dysfunctions in the muscular system may impact walking patterns [8, 21].

The static and dynamic balance in individuals with COPD was found to be significantly affected compared to non-COPD people. Posturography was used in the current study to evaluate static balance, a more objective measure in which individuals with COPD had impaired postural control in the eye-open, eye-closed condition compared to non-COPD individuals with increased sway velocity in anteroposterior (AP) and mediolateral (ML) and increased velocity moment (VM). Higher sway velocity and velocity moment is a sign of poor balance [15]. Findings are in the line with Almeida et al. [22] which noted individuals with COPD present balance deficits compared to healthy individuals. This can be explained by the rise in respiratory demand that is prevalent in people with COPD as it can impair balance due to increased activation of the trunk muscle [22, 23]. The results of the present study indicate that the balance in COPD individuals was not influenced by the severity of obstruction, as there was no significant correlation found between these two parameters statistically.

In our study, we observed that the TUG score is at the borderline in both groups. However, compared to non-COPD individuals, COPD individuals had increased timed up and go test scores, which may be due to reduced peripheral muscle strength leading to changes in dynamic balance [24]. These outcomes are in line with the study of Beauchamp et al. [25] and Chang et al. [26].

Fear of falling has been frequently identified as a risk factor for falls [27]. Fear of falls in COPD individuals was found to be greater than in non-COPD individuals in this study. In COPD individuals, the FES-I score was 32, indicating high concern about falling. Improved susceptibility to falls has been due to worsening of dyspnoea and poor health-related quality of life (HRQOL) [28].

The finding of this study illustrates the need to include the evaluation of static and dynamic balance, gait, and risk or fear of falls in the systematic assessment of patients with COPD for pulmonary rehabilitation. Future studies can focus on the study of the impact of a comprehensive rehabilitation programme, including balance training on functional outcomes and the quality of life associated with health in patients with COPD.

## 10. Conclusion

In the present study, individuals with COPD showed reduced swing time, reduced static and dynamic balance, and greater fear of falls than age- and gender-matched individuals with no history of COPD. These results will help to explain gait dynamics, balance, and fear of falls in people with COPD. Future studies should focus on investigating the impact of muscle fatigue, physical activity on gait dynamics, and postural control in individuals with COPD.

## Figures and Tables

**Table 1:** Demographic data of the subjects.

	COPD	Non-COPD Subjects	*p* value
*N*	42	45	
Age (mean ± S.D.)	61.90 ± 4.61	60.47 ± 6.18	0.16
BMI (mean ± S.D.)	22.22 ± 3.31	23.50 ± 3.27	0.11
Lung function			
FEV 1 (% predicted)	37.30 ± 7.99	—	
FVC (% predicted)	60.07 ± 10.58	—	
FEV1/FVC	61.30 ± 10.39	—	

COPD: chronic obstructive pulmonary disease.

**Table 2:** Comparison of gait parameters between COPD and Non-COPD subjects.

Gait parameters	COPD (*n* = 42)		Non-COPD (*n* = 45)		*p* value
	Median	Interquartile range	Median	Interquartile range	
Step duration (msec)	635.0	597.5, 702	610	515, 735	0.60
Gait cycle duration (msec)	1350	1195.0, 1477.5	1220	1120, 1365	0.10
Swing duration (msec)	1305	1220, 1385	1230	1110, 1350	0.044
Step length (mm)	545	500, 621	523	456, 593	0.23
Gait cycle length (mm)	960	904.5, 1087.7	950	832, 1054	0.24

**Table 3:** Comparison of static and dynamic balance between COPD and Non-COPD individuals.

Posturography parameters∗	COPD (*n* = 42)		Non-COPD (*n* = 45)		*p* value
	Median	Interquartile range	Median	Interquartile range	
Static balance					
NSEC-AP (mm/sec)	11.1	6.8, 16.7	7.1	5.2, 9.5	0.002
NSEC-ML (mm/sec)	7.0	4.8, 9.3	4.4	2.7, 6.95	<0.001
NSEC-VM (mm^2^/s)	12.9	11.8, 20.6	11.2	6.5, 20	0.11
NSEO-AP (mm/sec)	8.3	7.95, 9.65	7.1	4.70, 8.10	0.023
NSEO-ML (mm/sec)	5.1	3.8, 6.2	4.5	4.0, 5.8	0.213
NSEO-VM (mm^2^/s)	21.0	14.0, 26.3	9.9	5.8, 19.1	0.00964

Dynamic balance					
Timed up and go test time (sec)	13	12, 16	12	10, 12	<0.01

Fear of falls					
Falls Efficacy Scale score	32	24.75–36	24	19, 25	<0.01

NSEC-AP: normal standing eye-closed anteroposterior sway velocity. NSEC-ML: normal standing eye-closed mediolateral sway velocity. NSEC-VM: normal standing eye-closed velocity moment. NSEO-AP: normal standing eye-open anteroposterior sway velocity. NSEO-ML: normal standing eye-open mediolateral sway velocity. NSEO-VM: normal standing eye-open velocity moment.

**Table 4:** Spearman's rank correlation coefficient between Fev1% and balance parameter for COPD participants.

Balance parameter	*r*	*p* value
NSEC-AP (mm/sec)	0.14	0.39
NSEC-ML (mm/sec)	0.15	0.92
NSEC-VM (mm^2^/s)	0.25	0.10
NSEO-AP	0.23	0.80
NSEO-ML (mm/sec)	0.20	0.73
NSEO-VM	0.38	0.65

NSEC-AP: normal standing eye-closed anteroposterior sway velocity. NSEC-ML: normal standing eye-closed mediolateral sway velocity. NSEC-VM: normal standing eye-closed velocity moment. NSEO-AP: normal standing eye-open anteroposterior sway velocity. NSEO-ML: normal standing eye-open mediolateral sway velocity. NSEO-VM: normal standing eye-open velocity moment. No significant correlation observed between balance parameter and Fev1%.

## Data Availability

The data used to support the findings of this study are available from the corresponding author upon request.
